# Management of a Tubo-Ovarian Abscess: A Single-Centre Retrospective Cohort Study of Clinical Outcomes and Factors Associated With Failure of First-Line Medical Therapy

**DOI:** 10.7759/cureus.118009

**Published:** 2026-10-16

**Authors:** Aaya Satouf

**Affiliations:** 1 Obstetrics and Gynaecology, Leicester Royal Infirmary, Leicester, GBR

**Keywords:** antibiotic therapy, drainage, pelvic inflammatory disease, retrospective cohort, tubo-ovarian abscess

## Abstract

Objective: To describe the clinical characteristics, antibiotic management, and outcomes of a consecutive cohort of women admitted with tubo-ovarian abscess (TOA) and to determine the factors associated with failure of first-line medical therapy.

Design and setting: This is a retrospective cohort study, with the setting of the Gynaecology Department at the University Hospitals of Leicester, United Kingdom.

Population: A total of 61 women who had a working diagnosis of TOA were identified during the study period; 14 (23%) were excluded when an alternative final diagnosis was made, and a further three women were excluded due to incomplete outcome documentation, so 44 women with a confirmed diagnosis of TOA remained for analysis.

Methods: Demographic data, together with the microbiological, radiological, antibiotic, and procedural data, were systematically extracted from clinical records and coded. The outcomes were compared between women whose abscess resolved with antibiotics alone and those who required radiological or surgical drainage, using Fisher's exact test for categorical variables and the Mann-Whitney U test for continuous variables.

Main outcome measures: These are the success of first-line antibiotic therapy, need for image-guided or surgical drainage, and length of hospital stay (LOS).

Results: Median age was 41 years (IQR: 33.5-51), and median BMI was 28 kg/m² (IQR: 24-32.5). Among the 44 women with complete documented treatment outcomes, antibiotics alone were successful in 31 (70.5%), while 13 (29.5%) required further treatment with image-guided drainage or surgery. Procedures included image-guided drainage (n = 2), laparoscopic drainage or definitive laparoscopic surgery (n = 5), laparoscopic surgery converted to laparotomy (n = 3), and primary laparotomy (n = 3). A prior history of pelvic inflammatory disease (PID) (62% vs 3%, p < 0.001), previous admission with TOA (46% vs 13%, p = 0.043), and an abscess diameter >6.5 cm (64% vs 25%, p = 0.033) were each significantly more common among women whose medical management failed. LOS was also significantly longer in women who failed medical therapy (median 6 vs 4 days, p = 0.030). Almost a quarter (23%) of women initially coded as TOA (14/61 excluded cases) ultimately had an alternative diagnosis established on imaging, most commonly hydrosalpinx or salpingitis.

Conclusions: First-line antibiotic therapy resolved TOA in approximately 7 of 10 women with a documented treatment outcome in this cohort, which is in keeping with contemporary published series. Recurrent disease, a prior history of PID, and larger abscess diameter identify women at higher risk of failing medical therapy and may help target earlier senior/multidisciplinary review and consideration of early drainage.

## Introduction

Tubo-ovarian abscess (TOA) is an inflammatory mass involving the fallopian tube, ovary, and sometimes adjacent pelvic organs, and represents one of the most severe complications of pelvic inflammatory disease (PID). It develops in approximately one in three women admitted to hospital with PID and carries a risk of long-term complications, including infertility, chronic pelvic pain, and recurrent disease if not managed appropriately [1].

The optimal management strategy for TOA remains debated. Broad-spectrum intravenous antibiotics with anaerobic cover, as recommended by the Centers for Disease Control and Prevention (CDC) 2021 sexually transmitted infections treatment guidelines, are established as first-line therapy for most women, with drainage or surgery reserved for those who do not improve clinically [2]. Reported success rates for antibiotics alone vary widely across the literature, from as low as 16% to over 90%, with more recent large series reporting success in roughly 65-83% of cases [3-6]. Where medical therapy fails, image-guided (ultrasound- or CT-guided) drainage has been associated with the highest reported success rates and shortest hospital stays among minimally invasive approaches, followed by laparoscopic drainage, while conventional laparotomy is generally reserved for haemodynamically unstable women, ruptured abscesses, or cases where minimally invasive drainage is not realistically possible [3].

Factors associated with failed medical management proposed in the literature include larger abscess diameter, elevated inflammatory markers (C-reactive protein (CRP), white cell count), fever at presentation, bilateral or complex morphology, higher body mass index, and recurrent or previously treated TOA [4-8]. However, the discriminatory performance of existing clinical risk scores has generally been modest, and factors identified vary between cohorts, in part reflecting differences in case-mix, diagnostic thresholds, and local antibiotic policy [8,9].

This retrospective study was undertaken to characterise the presentation, the antibiotic regimen prescribed, and outcomes of TOA managed within the gynaecology assessment unit, and to determine whether readily available admission characteristics were associated with failure of first-line medical therapy. Alongside the clinical study, the diagnostic pathway was also examined, since a substantial minority of women referred with a working diagnosis of TOA were ultimately found to have an alternative diagnosis, which has implications for the interpretation of local TOA activity and resource planning.

## Materials and methods

Study design and setting

This was a retrospective cohort study of women admitted with a working diagnosis of TOA to the gynaecology assessment unit at Leicester Royal Infirmary over the study period of the 1st of December 2023 to the 1st of December 2024. 

Case identification and eligibility

Sixty-one women with a working diagnosis of TOA were identified for the study period. Fourteen women (23.0%) were subsequently excluded after review confirmed an alternative final diagnosis, most commonly hydrosalpinx, ectopic pregnancy, ovarian torsion, or endometritis (full breakdown in Table 1). The final diagnosis was reviewed using the available clinical documentation, radiological findings (including transvaginal ultrasound and CT reports), and operative findings, where applicable. A further three women had incomplete outcome documentation and were therefore excluded from the analysis. The remaining 44 women, in whom the diagnosis of TOA was confirmed on imaging and/or at surgery, constituted the analysis cohort.

**Table 1:** Reasons for exclusion of women initially referred with suspected tubo-ovarian abscess (n = 14).

Study no.	Final diagnosis	Hydrosalpinx clinically stable?	
1	Bilateral hydrosalpinx	Yes	
2	Surgical termination of pregnancy	N/A	
3	Bilateral hydrosalpinx	Yes	
4	Ectopic pregnancy	N/A	
5	Ovarian torsion	N/A	
6	Ovarian torsion	N/A	
7	Ectopic pregnancy	N/A	
8	Endometritis	N/A	
9	Paraovarian cyst / hydrosalpinx	Yes	
10	Hyperechoic mass, left adnexa	N/A	
11	Endometriosis	N/A	
12	Endometritis	N/A	
13	Ectopic pregnancy	Yes	
14	Endometriosis	Yes	

Data collection

Demographic data (age, body mass index (BMI)), pre-existing risk factors (history of PID, presence and duration of an intrauterine device (IUD/IUS), history of endometriosis, diabetes, postmenopausal status, recent hysteroscopy, fertility treatment, previous pelvic surgery, and previous TOA admission), admission clinical and inflammatory markers (fever, CRP), radiological features (abscess diameter, bilaterality/complexity), antibiotic regimen, duration of intravenous and total antibiotic therapy, use of image-guided (interventional radiology) drainage, surgical intervention (laparoscopic drainage, laparoscopic salpingo-oophorectomy/hysterectomy, or laparotomy), surgical specialties involved, and length of hospital stay (LOS) were extracted from clinical records into a structured dataset. Free-text entries were independently coded into binary or categorical variables; where documentation was ambiguous or missing, the variable was coded as missing rather than inferred, and denominators are reported explicitly for each analysis.

Treatment and outcome definitions

The primary outcome was success of first-line antibiotic therapy, defined as discharge without the need for image-guided or surgical drainage. Treatment outcome was explicitly documented for 44/47 women (93.6%). The three women without explicit outcome documentation were excluded from the treatment-outcome analysis rather than having their outcomes inferred. Missing information was not assumed to indicate treatment success or failure. Accordingly, comparative analyses of successful versus failed first-line medical management were restricted to the 44 women with documented outcomes.

Treatment failure requiring escalation was defined as the need for image-guided drainage or operative intervention, including laparoscopic drainage or definitive surgery, conversion to laparotomy, or primary laparotomy. The treatment outcome was classified according to whether invasive intervention was required during the index episode of care.

Patients were managed according to the treating team's clinical judgement and local practice, with antibiotic therapy as the initial treatment where clinically appropriate. Empirical antibiotic regimens included ceftriaxone with doxycycline and metronidazole, with treatment modified where indicated by clinical response and microbiological findings.

Antibiotic regimens, routes of administration, treatment modifications, and treatment durations were recorded from the available medical records where documented. The study was retrospective, and antibiotic choice and duration were determined by the treating team rather than by a predefined study protocol.

Statistical analysis

Continuous variables are presented as mean ± standard deviation or median (interquartile range (IQR)) according to distribution, and were compared between women with successful versus failed first-line medical management using the Mann-Whitney U test. Categorical variables are presented as n (%) and were compared using Fisher's exact test, appropriate given the small cell counts in this cohort. A two-sided p-value < 0.05 was considered statistically significant. Given the retrospective, hypothesis-generating nature of this study and the modest sample size, no correction for multiple comparisons was applied, and results should be interpreted as exploratory. Analyses were performed in Python 3.12.3 (pandas 3.0.2, numpy 2.4.4, scipy 1.17.1 (Python Software Foundation, Wilmington, DE) for Fisher' exact test and Mann-Whitney U).

Ethics approval

Formal ethics approval was not required for retrospective analysis of routinely collected, anonymised clinical data. No additional patient intervention or contact was undertaken for the purposes of the study 

Patient data and confidentiality

All patient-identifiable information (names and hospital numbers) was removed prior to analysis; patients are referred to only by anonymised study number throughout this manuscript and its underlying dataset.

## Results

Participant selection

During the study period, 61 women were identified with a working diagnosis of TOA. Fourteen women (23.0%) were excluded following review because an alternative final diagnosis was established.

Among the 47 women with confirmed TOA, treatment outcome was explicitly documented in 44 (93.6%). Of these, 31 women (70.5%) were successfully managed with antibiotics alone and 13 (29.5%) required image-guided or surgical intervention. The three women without complete outcome documentation were excluded from the treatment-outcome analysis.

Cohort characteristics

Forty-seven women had confirmed TOA. However, three of those women had incomplete outcome documentation and were excluded from further analysis. Median age was 41.0 years (IQR: 33.5-51.0; range: 20-74), and median BMI was 28.0 kg/m² (IQR: 24.0-32.5; range: 16-68).

Nine women (20.5%) had a previous history of PID, and 10 (22.7%) had previously been admitted with TOA. Eleven women (25%) were postmenopausal, and 14 (31.8%) had an IUD/IUS in situ at admission.

CRP was elevated (>25 mg/L) in 35/44 women (79.5%). Among the 35 women with a recorded CRP value, the median CRP was 198 mg/L (IQR: 138.5-234.5). An abscess diameter greater than 6.5 cm was documented in 14/39 women (35.8%) for whom the available imaging information permitted classification. Baseline demographic and clinical risk factors are summarised in Table 2.

**Table 2:** Baseline demographic and clinical characteristics of the cohort (N = 44). *Five women had ambiguous or alternative imaging descriptions (e.g., hydrosalpinx) precluding classification against this threshold. CRP: C-reactive protein; IQR: interquartile range; PID: pelvic inflammatory disease; TOA: tubo-ovarian abscess; IUD/IUS: intrauterine device

Characteristic	n / N	%
Age, years - median (IQR)	41.0 (33.5-51.0)	-
BMI, kg/m² - median (IQR)	28.0 (24.0-32.5)	-
Previous pelvic surgery	13/44	29.5
IUD/IUS in situ at admission	14/44	31.8
- Copper coil	8/44	18.2
- Mirena/levonorgestrel IUS	5/44	11.4
- Kyleena/other	1/44	2.3
Long-term IUD/IUS use	10/44	22.7
Previous admission with TOA	10/44	22.7
Postmenopausal	11/44	25
History of PID	9/44	20.5
Fever at admission	10/43	23.2
Endometriosis	8/44	18.2
Diabetes mellitus	5/44	11.4
Recent hysteroscopy	4/44	9.1
Fertility treatment	2/44	4.5
CRP raised (>25 mg/L)	35/44	79.5
CRP value, mg/L - median (IQR), n=34	198 (138.5-234.5)	-
Abscess diameter >6.5 cm on imaging*	14/39	35.8
Bilateral and/or complex morphology	17/44	38.6

Antibiotic management

The most commonly documented antibiotic approach was a ceftriaxone and metronidazole-doxycycline-based regimen, used in 29/44 women (65.9%), frequently with intramuscular ceftriaxone, intravenous metronidazole, and oral doxycycline, followed by oral step-down therapy. Co-amoxiclav-containing regimens were documented in 4/44 women (9.1%), while piperacillin-tazobactam (Tazocin)-containing regimens were used in 3/44 (6.8%). Gentamicin plus clindamycin, primarily in the context of penicillin allergy, was used in 2/44 women (4.5%). Two women (4.5%) received complex broad-spectrum escalation regimens, while a fluoroquinolone-containing regimen was documented in 1/44 (2.3%). Overall, antibiotic management frequently involved sequential intravenous and oral therapy, with escalation or modification according to clinical circumstances. The median planned duration of the initial antibiotic course was 14 days (IQR: 8.25-14.0), consistent with standard PID/TOA treatment protocols [1].

Table 3 shows the antibiotic management regimens used for treatment.

**Table 3:** Antibiotic management regimens used.

Antibiotic management	n	%
Ceftriaxone + Metronidazole + doxycycline-based regimen	29	65.9
Co-amoxiclav-containing regimen	4	9.1
Piperacillin-tazobactam (Tazocin)-containing regimen	3	6.8
Gentamicin + clindamycin (penicillin allergy)	2	4.5
Broad-spectrum escalation/combination regimen	2	4.5
Fluoroquinolone-containing regimen	1	2.3
Other regimen	2	4.5
No antibiotic regimen documented	1	2.3
Total	44	100

Outcome of first-line therapy and management pathway

Outcomes were explicitly documented for 44/47 women (93.6%): antibiotics alone were successful in 31 (70.5%) and failed in 13 (29.5%).

Thirteen women (29.5%) underwent an invasive procedure: image-guided (interventional radiology) drainage alone in two (4.5%); laparoscopic drainage or definitive laparoscopic surgery (e.g., laparoscopic total hysterectomy and bilateral salpingo-oophorectomy) in five (11.4%); laparoscopic surgery converted to laparotomy in three (6.8%); and primary laparotomy in three (6.8%). The overall management pathway is shown in Figure 1.

**Figure 1:**
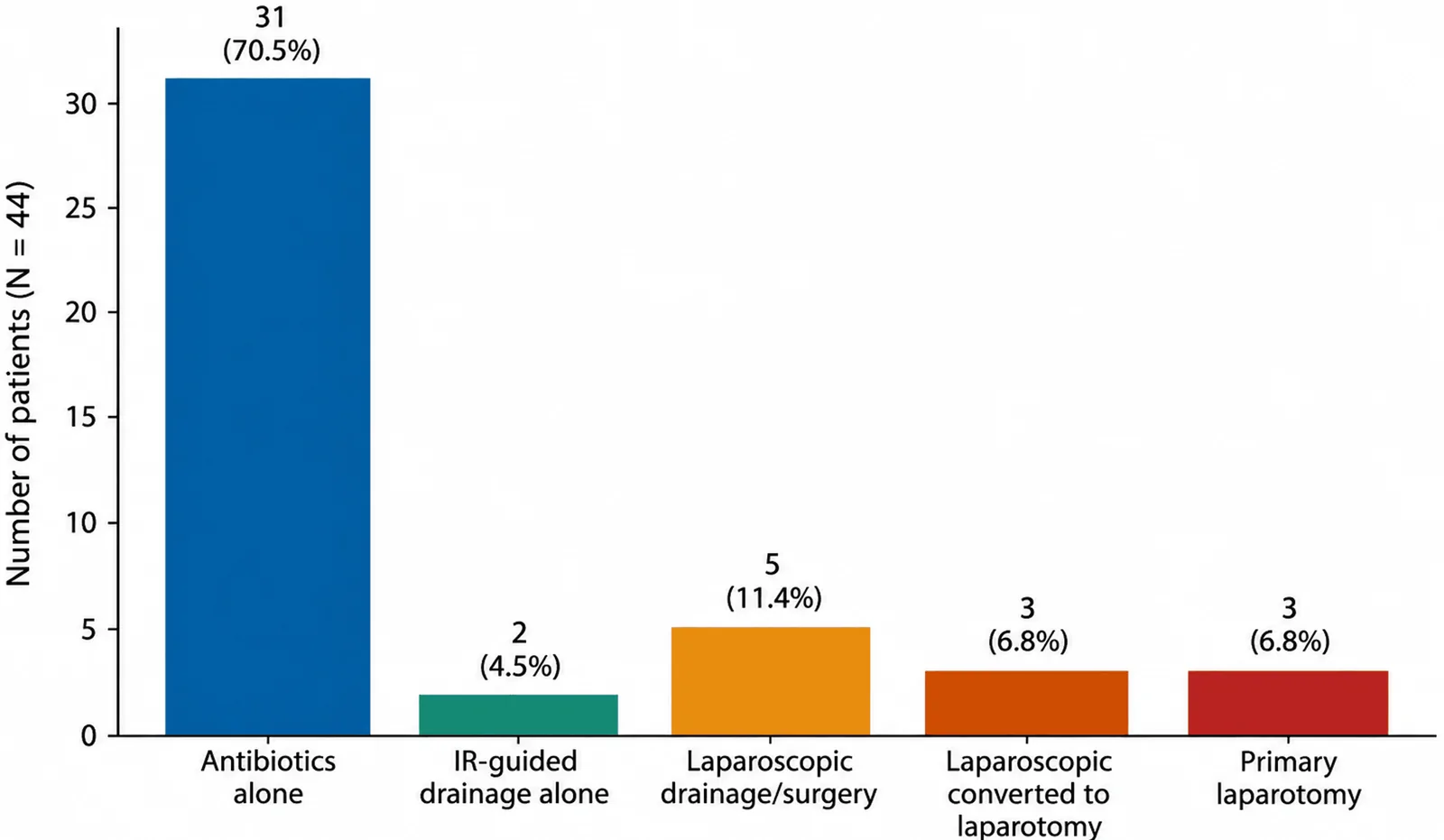
Management pathway of the tubo-ovarian abscess cohort (N = 44).

Diagnostic uncertainty

Fourteen women originally referred with suspected TOA were excluded from the analysis cohort after an alternative final diagnosis was confirmed - most commonly hydrosalpinx, ectopic pregnancy, ovarian torsion, or endometritis. Together, these findings indicate that a substantial proportion of women referred with suspected TOA (22.9% of the total 61 women initially identified) did not have straightforward, unambiguous TOA on imaging.

Factors associated with failed first-line medical management

Table 4 compares baseline and admission characteristics between the 31 women whose abscess resolved with antibiotics alone and the 13 women whose medical management failed (analysis restricted to the 44 women with an explicitly documented outcome).

**Table 4:** Comparison of clinical characteristics between women with successful versus failed first-line antibiotic therapy (N = 44 with documented outcome). Fisher's exact test for categorical variables and the Mann-Whitney U test for continuous variables. Significant associations (p < 0.05) were history of PID, previous TOA admission, abscess diameter >6.5 cm, and length of stay. CRP: C-reactive protein; IQR: interquartile range; PID: pelvic inflammatory disease; TOA: tubo-ovarian abscess; IUD/IUS: intrauterine device

Characteristic	Success (n=31)	Failure (n=13)	p-value
History of PID	1/31 (3%)	8/13 (62%)	<0.001
Previous TOA admission	4/31 (13%)	6/13 (46%)	0.043
Abscess diameter >6.5 cm	7/28 (25%)	7/11 (64%)	0.033
Fever at admission	5/31 (16%)	5/12 (42%)	0.11
Postmenopausal	10/31 (32%)	1/13 (8%)	0.132
Bilateral/complex mass	10/31 (32%)	7/13 (54%)	0.309
Endometriosis	5/31 (16%)	3/13 (23%)	0.676
Previous pelvic surgery	10/31 (32%)	3/13 (23%)	0.722
IUD/IUS in situ	11/31 (35%)	3/13 (23%)	0.498
Diabetes mellitus	4/31 (13%)	1/13 (8%)	1
CRP raised (>25 mg/L)	25/31 (81%)	10/13 (77%)	1
Age, years - median (IQR)	45.0 (34.5-52.5)	38.0 (31.0-45.0)	0.172 U=148.0
BMI, kg/m² - median (IQR)	28.0 (24.0-34.0)	26.0 (23.0-28.0)	0.290 U=160.0
CRP, mg/L - median (IQR)	196 (154-227)	200 (101-269)	1.000 U=113.0
Length of stay, days - median (IQR)	4.0 (2.0-5.0)	6.0 (4.0-11.0)	0.030 U=117.5

A history of PID was more common among women whose medical treatment failed than among those successfully treated with antibiotics alone (8/13 (61.5%) vs 1/31 (3.2%), p < 0.001). Previous admission with TOA was also more common among women with treatment failure (6/13 (46.2%) vs 4/31 (12.9%), p = 0.043).

An abscess diameter >6.5 cm was present in 7/11 (63.6%) women with treatment failure compared with 7/28 (25.0%) women whose medical treatment was successful (p = 0.033).

Fever at admission was more frequent among women with treatment failure than successful treatment (5/12 (41.7%) vs 5/31 (16.1%)), although this difference was not statistically significant (p = 0.110). Similarly, bilateral and/or complex morphology was more common in the treatment-failure group (7/13 (53.8%) vs 10/31 (32.3%)), but the difference was not statistically significant (p = 0.309). This is demonstrated by Figure 2.

**Figure 2:**
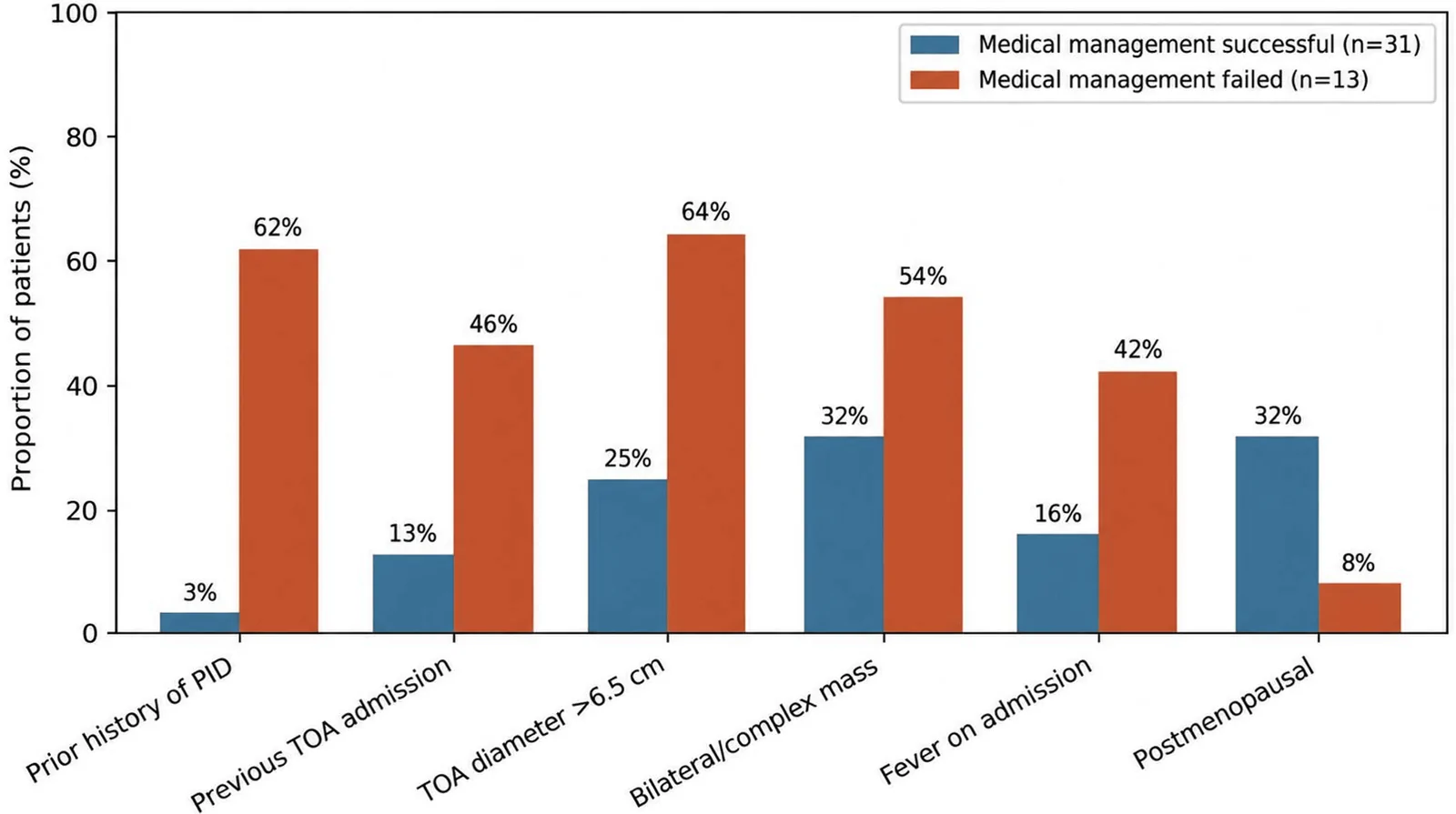
Prevalence of selected clinical characteristics among women whose first-line antibiotic therapy succeeded versus failed. The numbers were rounded to the nearest whole percentage point.

No statistically significant differences were identified for postmenopausal status, endometriosis, previous pelvic surgery, IUD/IUS use, diabetes, or raised CRP.

Age was similar between groups (median: 45.0 vs 38.0 years; Mann-Whitney U = 148.0, p = 0.172). BMI was also similar (U = 160.0, p = 0.290). CRP levels did not differ (U = 113.0, p = 1.000). LOS was significantly longer in the failure group (U = 117.5, p = 0.030).

Because only 13 women had explicitly documented treatment failure, no multivariable model was fitted. The observed associations should therefore be interpreted as unadjusted associations and not as independent predictors.

Length of stay

Overall median LOS was four days (IQR: 3-7; range: 1-40). Among women with documented treatment outcomes, those who required escalation to image-guided or surgical drainage had a significantly longer LOS than those managed with antibiotics alone (median 6 vs 4 days, p = 0.030). This comparison included the 13 women who underwent an invasive procedure and the 31 women successfully managed with antibiotics alone. Table 5 demonstrates the difference in LOS between patients treated with antibiotics alone compared with patients who underwent invasive intervention.

**Table 5:** Length of stay in patients treated by antibiotics alone in comparison with patients that underwent invasive intervention. The longest admission was 40 days, involving a woman managed with a prolonged six-week course of outpatient parenteral antibiotic therapy (OPAT) for a large, complex abscess without invasive drainage.

Length of stay (days)	Overall (N=44)	Antibiotics alone (n=31)	Invasive intervention (n=13)
Median (IQR)	4 (2.75-6.25)	4 (2-5)	6 (4-13.5)
Range	1-40	1-40	2-23

The single longest admission (40 days) was related to a woman managed with a prolonged six-week course of outpatient parenteral antibiotic therapy (OPAT) for a large, complex abscess without invasive drainage.

## Discussion

Principal findings

In this cohort of 44 women with confirmed TOA and explicit treatment outcome documentation, first-line antibiotic therapy alone was successful in 31 (70.5%), broadly consistent with several contemporary published series reporting success rates of 65-83% [5,6]. Our success rate was somewhat higher than the 56.5% reported by Teh et al. [7] and closer to the 65.1% reported in the larger TOAST cohort [6] and the 81.6% reported in an Asian tertiary-centre series by Chan et al. [5], which likely reflects differences in case-mix, abscess severity at presentation, and local thresholds for early drainage.

A prior history of PID, a previous admission with TOA, and an abscess diameter greater than 6.5 cm were each significantly associated with failure of first-line medical therapy. These findings are broadly consistent with previously published predictors of treatment failure: the TOAST study identified fever at admission, larger abscess diameter, and higher CRP as independent predictors of failure [6]; Chan et al. identified increasing abscess size and BMI as independent predictors in a multivariable model, proposing a size threshold of approximately 7.4 cm [5]; and DeWitt et al. demonstrated that larger abscesses were associated with longer hospitalisation and greater need for surgical intervention [4]. Systematic review and meta-analysis evidence has also identified several clinical and inflammatory factors associated with failure of antibiotic therapy [8].

Our identification of previous PID and previous TOA admission as strong factors associated with failed first-line medical therapy - with the largest effect size in this cohort - may reflect a distinct, recurrent phenotype of tubal disease with more extensive adhesions, fibrosis, and reduced antibiotic penetration, making these women less likely to respond to a further course of medical therapy alone. This has not been consistently highlighted in prior series and may be a useful, low-cost, readily available flag at the point of admission. Unlike some larger series, we did not find statistically significant associations between failure of medical management and fever, CRP, BMI, or bilateral/complex morphology. This most likely reflects the limited statistical power of a 44-patient comparison rather than a true absence of association; the point estimates for fever (42% vs 16%) and bilateral/complex disease (54% vs 32%) were directionally consistent with the wider literature, and larger prospective or multicentre data would be needed to confirm or refute these associations locally [5,6,8].

Antibiotic prescribing

Just over half of women received a ceftriaxone, doxycycline, and metronidazole-based regimen in line with CDC 2021 guidance for PID complicated by TOA, which recommends ceftriaxone with doxycycline and metronidazole for anaerobic cover [2]. This finding supports the role of broad-spectrum antimicrobial therapy as an appropriate initial approach in selected patients with TOA. Importantly, antibiotic selection should provide adequate coverage against the polymicrobial spectrum of TOA, including anaerobic organisms and sexually transmitted pathogens, while treatment should subsequently be tailored according to microbiological results and clinical response when available. The relatively high proportion of patients responding to antibiotics alone in our cohort suggests that early conservative management may be effective in appropriately selected patients, although prospective studies are required to better define clinical and radiological criteria that can predict failure of antibiotic therapy and the need for drainage.

Escalation pathway and LOS

Just over a quarter of women required escalation beyond antibiotics alone, most often to laparoscopic drainage or surgery; only two women (4.3%) underwent image-guided drainage alone. This is a lower rate of interventional radiology use than reported in some other series and systematic reviews, which have highlighted image-guided drainage as achieving the highest success rates and shortest LOS among invasive options [3]. Given that women who required any escalation in our cohort had a significantly longer LOS, increasing access to early image-guided drainage - where an appropriate target is identified, and interventional radiology capacity allows - may represent an opportunity to reduce both bed-days and the need for surgery in appropriately selected women who are failing to improve on antibiotics.

Diagnostic uncertainty

A notable secondary finding was the frequency of diagnostic uncertainty: 14 of 61 women (23%) referred with suspected TOA had an entirely different final diagnosis (most commonly hydrosalpinx, ectopic pregnancy, ovarian torsion, or endometritis). This underlines the recognised difficulty in distinguishing TOA from other adnexal pathology on ultrasound alone, particularly early or partially treated disease, and supports a low threshold for repeat or cross-sectional imaging and senior gynaecological review where the diagnosis is unclear, given the differing management implications (for example, ectopic pregnancy and ovarian torsion are time-critical surgical emergencies distinct from TOA).

Strengths and limitations

This study has several strengths: it captures granular, patient-level detail on risk factors, antibiotic choice, and the full escalation pathway rather than relying on coded administrative data alone, and it explicitly reports diagnostic uncertainty and missing data rather than assuming complete ascertainment.

Its principal limitation is sample size: with 47 women in the overall cohort and only 44 with a documented primary outcome, the comparative analysis in Table 4 is underpowered to detect all but the strongest associations, confidence intervals around the reported proportions are wide, and no multivariable adjustment for confounding was possible. The retrospective, single-centre design is subject to documentation bias, and several variables (notably ESR, which was almost never recorded, and exact abscess dimensions in women with alternative imaging descriptions) had substantial missing data. Three women with confirmed TOA lacked complete treatment-outcome documentation and were therefore excluded from the analysis. We did not capture long-term reproductive or fertility outcomes, recurrence beyond the index admission, or formal complication/readmission data, all of which would strengthen future work. Finally, as with any single-centre series, local antibiotic policy, referral patterns, and threshold for drainage limit the generalisability of absolute success and escalation rates to other settings, although the identified factors associated with treatment failure are conceptually consistent with larger published cohorts [5-8].

Implications for practice and future work

These findings support continuing to offer a trial of first-line broad-spectrum intravenous antibiotics to the majority of haemodynamically stable women with TOA, consistent with current national guidance [2], while flagging women with a prior history of PID, a previous TOA admission, or a larger abscess (>6.5 cm) for closer monitoring, earlier senior review, and earlier consideration of image-guided or surgical drainage if early clinical response is not seen. A prospective, adequately powered multicentre study - ideally incorporating a standardised risk-prediction tool such as that proposed by Yongue et al. [9] or validated in the TOAST cohort [6] - would help confirm these associations and support the development of a locally validated escalation protocol. Given the frequency of diagnostic uncertainty identified here, a parallel focus on standardising ultrasound reporting criteria for TOA versus its principal differential diagnoses would also be of value.

## Conclusions

In this retrospective cohort, treatment outcomes were documented in 44 women with confirmed TOA, of whom approximately seven out of 10 were successfully treated with first-line antibiotics without the need for drainage or surgery. This is consistent with contemporary published outcomes. A prior history of PID, previous TOA admission, and larger abscess diameter were each associated with failure of medical management and with longer hospital stay. These characteristics may help identify, at the point of admission, women who would benefit from closer inpatient monitoring and earlier consideration of drainage, while a substantial minority of women referred with suspected TOA were found to have an alternative diagnosis, reinforcing the importance of diagnostic vigilance in this presentation.
